# Pollen and Phytolith Evidence for Rice Cultivation and Vegetation Change during the Mid-Late Holocene at the Jiangli Site, Suzhou, East China

**DOI:** 10.1371/journal.pone.0086816

**Published:** 2014-01-23

**Authors:** Zhenwei Qiu, Hongen Jiang, Jinlong Ding, Yaowu Hu, Xue Shang

**Affiliations:** 1 Key Laboratory of Vertebrate Evolution and Human Origins of Chinese Academy of Sciences, Institute of Vertebrate Paleontology and Paleoanthropology, Chinese Academy of Sciences, Beijing, China; 2 Department of Scientific History and Archaeometry, University of Chinese Academy of Sciences, Beijing, China; 3 Suzhou Institute of Archaeology, Suzhou, China; Manchester Institute of Biotechnology, United Kingdom

## Abstract

Pollen and phytolith analyses were undertaken at the Jiangli site in Suzhou, Jiangsu Province, combined with studies on macrofossils by flotation. The concentration of pollen decreased while the percentage of Poaceae pollen in the profile increased from the late phase of the Majiabang Culture to the Songze Culture suggesting that human impact on the local environment intensified gradually. The discovery of rice paddy implies a relatively advanced rice cultivation in this area during the middle-late Holocene. Other than phytoliths, the high percentage of *Oryza*-type Poaceae pollen (larger than 40 µm) supplied robust evidence for the existence of rice paddy. Moreover, the fact that the farther from the rice paddy, the lower the concentration and percentage of Poaceae pollen also proves that the dispersal and deposition of pollen is inversely proportional to the distance.

## Introduction

Microfossils, especially pollen (and spores) and phytoliths, which are reproductive bodies and cell linings of land plants, can provide many important indications about the evolution of the natural world and human society. Pollen analysis has an important role to play in the reconstruction of the paleoenvironment and landscape of an archaeological site and the surrounding areas [1]–[5]. NPM (non-pollen microfossils) including phytoliths [6], diatoms [7] and starch grains [8], [9] play a necessary complementary role. It is noted that, being produced primarily by local vegetation, phytoliths reflect local plant assemblages [10]. Besides, macrofossils, usually obtained by flotation, shed light on paleoclimate and paleobotany [11]–[13].

Research on the origin and development of rice agriculture has yet to reach a consensus, but in general rice domestication and cultivation is considered to have originated in the middle and lower reaches of the Yangtze River [14]–[16] at least by the late phase of the Majiabang Culture (cal. 6500 BP); in other words, rice cultivation technology had been proficiently mastered by Neolithic people [13], [17]–[24]. To date, research on rice paddies excavated in China and Japan always showed a very high concentration of *Oryza*-type bulliform phytoliths in archaeological samples. In view of its limitations, pollen studies, however, neglected paleo-agricultural analysis. Nevertheless, some attempt to distinguish cereal from grass pollen grains [25] was used to investigate the paleo-agriculture of special areas, such as the lower reaches of the Yangtze River [26].

### Natural Environment and Archaeological Context

**Figure 1:**
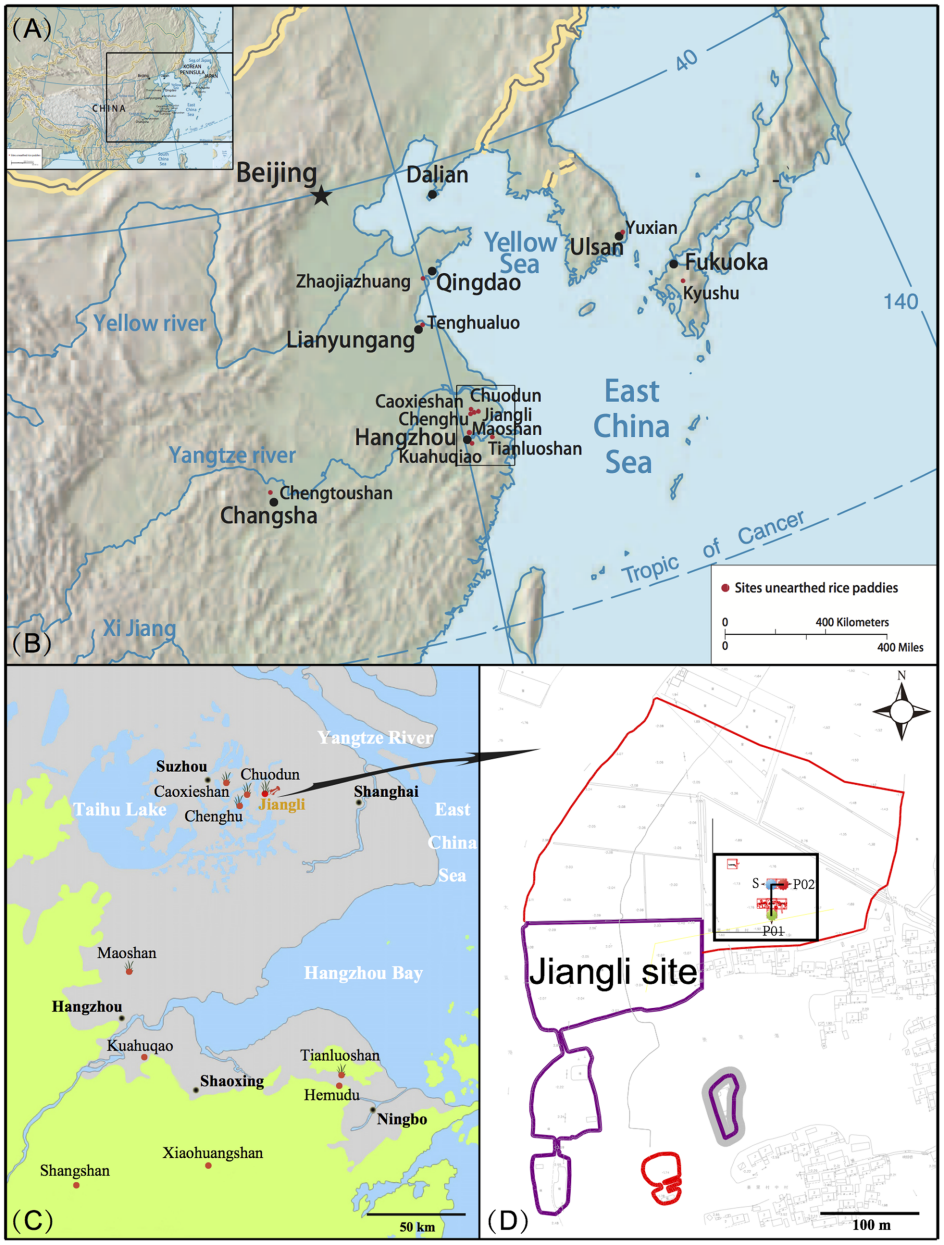
Location of the Jiangli site. (A) Heartlands of ancient East Asian rice farming; (B) Archaeological sites where ancient rice paddies have been unearthed in East Asia; (C) Archaeological sites related to rice agriculture in the lower reaches of the Yangtze River; (D) Sampling locations at the Jiangli site.

Jiangli site (31°15′1.80″ N; 120°55′4.44″ E; 1.80 m ASL) is located in a small basin surrounded by low mountains and hills. Situated in Jiangli Village, Kunshan City, to east of Taihu Lake, the archaeological site is located on the East Lake Island and the West Lake Island. It is north of Dongyuemiao and Houcun, east of Dazhigang and west of Dalou, covering a total area of about 90,000 square meters (Fig. 1).

Taihu Lake, China’s third largest freshwater lake, is located in the Yangtze River Delta (30°56′–31°34′ N; 119°54′–120°36′E). It covers an area of 2428 km^2^, with an average depth of 1.89 m, which makes it a typical plain shallow lake. This area is influenced by the subtropical monsoon, having four distinct seasons, with a moderate and moist climate. The average temperature is 15–17°C, annual precipitation is 1000–1400 mm, and the frost-free period lasts for 220–246 days [27], [28].

Today the area is mostly occupied by cultivated vegetation, especially rice (*Oryza sativa*) [29]. Northern subtropical mixed evergreen and deciduous broad-leaved secondary or successional forests (*Castanopsis*, *Quercus*, *Betula*, and *Liquidambar* are the most representative and dominant species) present on isolated hills amid the Yangtze River Delta plain and on mountains flanking the east and south of the area as well [27], [30].

**Figure 2:**
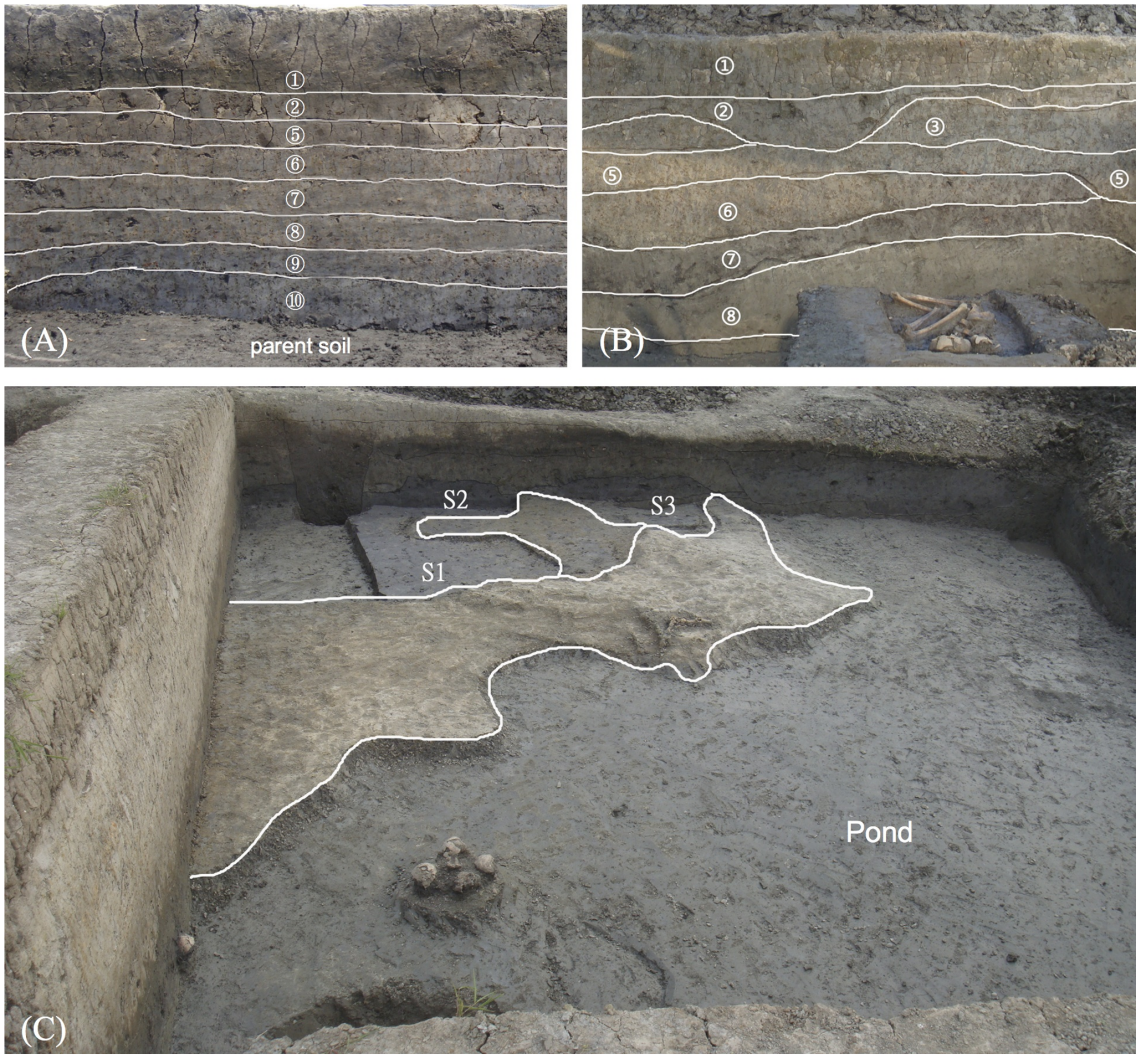
Photos and sketches of sampling locations at the Jiangli site. (A) Profile 01; (B) Profile 02; (C) Rice paddies (S1–S3).

The cultural deposits in the central area of the site are more than 2 meters thick, which from the bottom up exhibited a sequence comprising cultures of the Majiabang, Songze, Liangzhu, Maqiao, the Han Dynasty, the Southern and Northern Dynasties and the Song Dynasty. The Suzhou Institute of Archaeology carried out a salvage excavation [31] from July to September 2011, directed by Prof. Jinglong Ding, one of the co-authors of the present paper. The excavated area is 600 square meters in total, which covered cemeteries of the Songze and Liangzhu Cultures, dwellings of Majiabang and Songze Cultures, and artifacts (including pottery, stone tools, jades, etc.). More importantly, a river-channel dating from the Majiabang Culture, together with rice paddies (S1–S3) and ponds (Fig. 2) of the Songze Culture were unearthed. It is noted that the Jiangli site is close to the Caoxieshan, Chuodun and Chenghu sites, all of which had Late Neolithic rice paddies.

## Materials and Methods

### Ethics Statement

All necessary permits were obtained from the Suzhou Institute of Archaeology for the described field studies.

### Material

Based on systematic and typical sampling principles [13], 17 sedimentary samples (Table 1) including two profiles (P01&P02), three paddy fields (S1–S3) (Fig. 2), and other deposits for analysis of seeds, phytoliths and pollen were taken from 6 trenches distributed from Location I to III at the Jiangli site. The samples from these 2 profiles were continuously taken layer by layer except for the surface. The average amount of each sample was about 4 L, of which nearly 3.5 L was used for flotation in order to obtain seeds and fruits.

**Table 1:** Morphological description of sedimentary samples from the Jiangli site.

Profile	Deposits	Depth (cm)	Description of samples				Cultural attributes
			Color	Structure	Texture	Inclusions	
	TE01N01 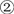	75–85	gray-brown	soft-porous	Clay	Celadon sherd, particlesof burned soil	Song Dynasty
	TE01N01 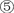	80–94	dark brown	hard-compact	Clay	Pottery sherd, particlesof burned soil	Songze Culture
	TE01N01 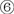	115–130	dark gray	hard-compact	Clay	Pottery sherd, particlesof burned soil	Songze Culture
**P01**	TE01N01 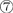	135–142	gray	hard-compact	Clay	Pottery sherd, particlesof burned soil	Songze Culture
	TE01N01 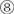	145–155	brown	hard-compact	Clay	Pottery sherd, particlesof burned soil	Songze Culture
	TE01N02 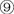	155–170	gray-green	hard-compact	Clay	Pottery sherd, particlesof burned soil	Majiabang Culture
	TE01N01 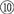	165–180	black	hard-compact	Clay	Pottery sherd, charcoal	Majiabang Culture
	TE01N01 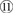	>185	yellow	hard-compact	Clay	Some Fe & Mnconcretion	parent soil
	TE02N04 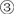	60	gray-brown	hard-compact	Clay	Pottery sherd, particlesof burned soil	Songze Culture
	TE02N04 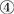 *	80	yellow-brown	hard-compact	Clay	Pottery sherd, particlesof burned soil	Songze Culture
**P02**	TE02N04 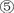	110	gray-brown	hard-compact	Clay	Pottery sherd, particlesof burned soil	Songze Culture
	TE02N04 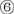	150	dark brown	hard-compact	Clay	Pottery sherd	Songze Culture
	TE02N04 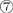	165	light gray	hard-compact	Clay	Pottery sherd	Songze Culture
	TE02N04 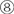	180	gray-brown	soft-porous	Clay	Pottery sherd	Majiabang Culture
	S1 below TE01N04 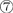		gray	friable-minute	Clay		Songze Culture
**Rice paddy**	S2 below TE01N04 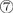		gray	friable-minute	Clay		Songze Culture
	S3 below TE01N04 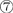		gray	friable-minute	Clay		Songze Culture

Samples of TE02N04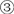-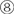 were collected from the East wall of TE02N04, except for TE02N04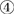 that was from the West wall.

### Methods

#### Dating

Charred rice grains, *Polygonum aviculare* seeds and charcoal were selected from carbonized seeds and charcoals retrieved by flotation, and dated by accelerator mass spectrometer (AMS) ^14^C at Peking University, then calibrated using IntCal04 [32] and OxCal v3.10 [33].

#### Sample preparation

Given that these sedimentary samples consist of compact clay, they need to be prepared prior to phytolith and pollen analyses. The samples were dried in a convection oven (DHG-9420A) at 100°C for 24 hours and then ground into powder in a mortar. Finally, the powder samples were packaged individually and placed in sealed boxes to prevent contamination.

#### Pollen analysis

Palynological analysis was carried out on the basis of procedures put forward by Moore et al. [34], Lentfer et al. [35] and Horrocks [36]. 50 g dried powder samples were processed with HCl, KOH (10%, 100 ml), ZnCl_2_ (2.0 g/ml in density, 30 ml), acetic acid (100 ml), KI/IH (2.0 g/ml in density, 5 ml) and acetolysis mixture (1 ml concentrated H_2_SO_4_, 9 ml acetic anhydride). A tablet of *Lycopodium* marker (18583 grains) was added to each sample. The pollen samples were spread uniformly on glass slides and at least 500 pollen grains, excluding aquatic pollen and spores, identificated and counted using a Nikon Eclipse LV100POL microscope. Identification was aided by the use of reference materials collected by the Key Laboratory of Vertebrate Evolution and Human Origins of Chinese Academy of Sciences, Institute of Vertebrate Paleontology and Paleoanthropology, Chinese Academy of Sciences and published keys [37].

#### Phytolith analysis

Following Lentfer and Boyd [38] and Pearsall [39], each 5 g dried powder sample was processed with NaHCO_3_ (5%, 30 ml), HCl (10%, 30 ml), H_2_O_2_ (30%, 30 ml) and KI/CdI_2_ (2.3 g/ml in density). In order to remove particles less than 20 µm, the sample was sedimented by Stokes’ Law. Based on a total of at least 500 phytoliths, identification and counting were carried out after the sample was dried at room temperature. Identification was made under a Nikon Eclipse LV100POL microscope using the standard keys [10], [39], [40]–[43] and the reference collection of the Key Laboratory of Vertebrate Evolution and Human Origins of Chinese Academy of Sciences, Institute of Vertebrate Paleontology and Paleoanthropology, Chinese Academy of Sciences. The phytoliths were designated in accordance with the International Code for Phytolith Nomenclature [44].

#### The climate index analysis

The climate index [41], [45] is an index which can be used to indicate warm or cold by phytolith morphotypes. In this case, warm type phytoliths include bulliforms, long-saddles, square and rectangular short cells, while cool type phytoliths contain short-saddles, trapeziform sinuate, rondel, acicular hair cells and elongate long cells.

The formula is as follows:
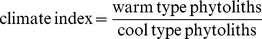



## Results

### Dating

According to analyses of archaeological typology and cultural characteristics, the ages of the deposits from TE01N04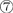, TE01N01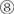 and S1 (the No.1 rice paddy) are from the Songze Culture (4000 BC-3300 BC), while that of M12 (a tomb with human skeleton, pottery and charcoals) belongs to the Liangzhu Culture (3300 BC-2200 BC). Besides, deposits and cultural remains from TE01N02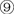, TE01N01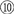, TE02N04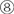 and TW01N02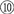 are considered to be from the Majiabang Culture (5000–4000 BC).

The AMS dates obtained from seeds and charcoals range from 3600 BC to 3000 BC, which is in good agreement with the date-range of the Songze Culture to the Liangzhu Culture (Table 2). In particular, the dating of *Polygonum aviculare* seeds from S1 is about 3600 BC-3500 BC, which belongs to the late stage of the Songze Culture. Considering S1, S2, S3 (Fig. 2) are close to each other and are all from the same layer which is below the deposit of TE01N04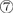, the dating of S2 and S3 should be similar to that of S1, i.e. the late Songze Culture.

**Table 2:** Results of radiocarbon dating: age of the ancient seeds and charcoal recovered.

Sample NO.	Sample	DepositionalUnit	^14^C years(T_1/2_ = 5568)	Dendrocalibrated AgeRanges (±1δ, 68.2%)	Dendrocalibrated AgeRanges (±2δ, 95.4%)
	Charred			3340–3310 BCE (10.6%)	
BA111366	rice grains	TE01N04 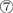	4510±30	3300–3260 BCE (3.9%)	3360–3090 BCE (95.4%)
				3240–3110 BCE (53.7%)	
				3320–3230 BCE (25.5%)	3320–3230 BCE (25.5%)
BA111367	Charcoal	M12	4445±30	3170–3160 BCE (1.2%)	3190–3150 BCE (6.2%)
				3110–3020 BCE (41.5%)	3140–3000 BCE (48.8%)
					2990–2930 BCE (3.7%)
				3630–3600 BCE (7.5%)	3630–3580 BCE (16.9%)
BA121676	Charred rice grains	TE01N01 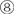	4700±30	3530–3490 BCE (16.1%)	3540–3370 BCE (78.5%)
				3450–3370 BCE (44.6%)	
BA121677	*Polygonum* *aviculare* seeds	S1 (belowTE01N04 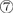 )	4810±30	3650–3630 BCE (17.3%)	3660–3620 BCE (25.2%)
				3580–3530 BCE (50.9%)	3600–3520 BCE (70.2%)

### Pollen Assemblages and Phytolith Record

**Figure 3:**
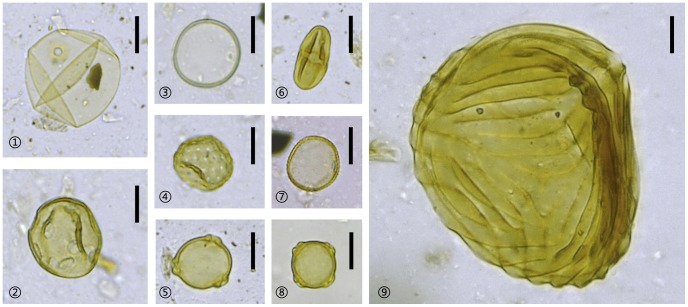
Pollen and spores from the Jiangli site. 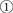
 Poaceae (>40 µm); 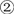 *Liquidambar*; 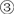 Poaceae (<40 µm); 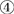 Chenopodiaceae; 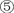 *Betula*; 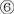 *Castanopsis*; 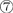 *Typha*; 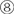 *Myriophyllum*; <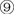 *Ceratopteris*. Scales  = 20 µm.

**Figure 4:**
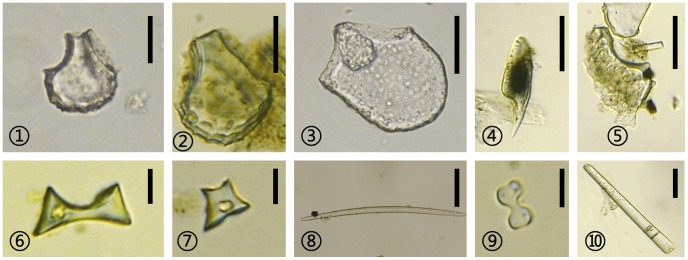
Phytoliths from the Jiangli site. 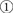
,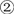 *Oryza*-type bulliform; 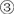 Reed-type bulliform; 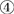 Acicular hair cell; 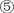 Double-peaked *Oryza*-type; 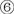 Bamboo-saddle; 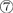 Short-saddle; 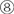 Sponge spicule; 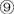 Bilobate; 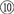 Elongate psilate long cells. Scales: 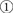,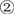, 20 µm; 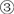,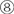, 40 µm; 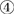, 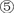, 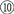, 50 µm; 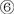,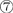,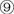, 10 µm.

Figures 3 and 4 demonstrate main morphotypes of pollen and phytoliths extracted from these sedimentary samples.

#### Paleoecological data of P01

**Figure 5:**
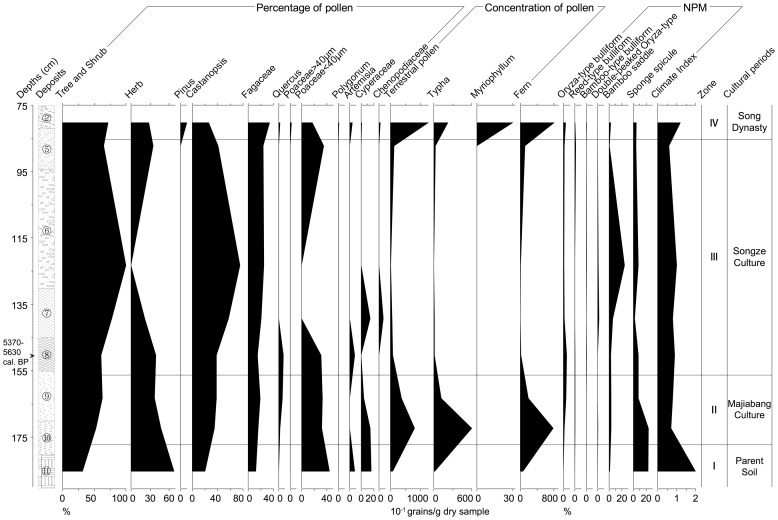
Integrated paleoecological data of P01 from the Jiangli site.

The pollen concentrations and percentages of major taxa provide a basis for dividing the diagram into four pollen zones (Fig. 5):

Zone 1 (before 7000 cal. BP, below 180 cm in depth).

The concentration of total terrestrial pollen is rather low in this zone. The pollen is dominated by terrestrial herbs (up to 68%), such as Poaceae, Cyperaceae, and *Artemisia*, among which mainly grass-type Poaceae pollen (grain diameter <40 µm) that reaches 44%. Besides, *Castanopsis* and other Fagaceae occur in relatively high percentages (up to 20% and 12% respectively). Furthermore, concentrations of aquatic herb pollen (e.g. *Typha*) and fern (e.g. *Ceratopteris*, *Pteris*) spores are rather low, which is in coherence with the situation of arboreal pollen.

Zone 2 (about 6000–7000 cal. BP, 155–180 cm in depth).

The concentration of total terrestrial pollen is rather high in this zone. The pollen is dominated by trees (up to 63%), mainly *Castanopsis* and *Quercus*, among which the average percentage of *Castanopsis* is above 35%. While there is a rather high percentage of grass pollen (32% or more). Meantime, concentrations of aquatic herb pollen and fern spores reach their highest value, i.e. nearly 60 and 70 grains per gram dry sample respectively. The majority of these are *Typha* pollen and trilete spores.

Zone 3 (5300–6000 cal. BP, 80–155 cm in depth).

The concentration of total terrestrial pollen is the lowest in this zone. The pollen is dominated by trees (up to 100%), including *Castanopsis* and *Quercus*. The percentages of *Castanopsis* pollen vary from 38% to 75% (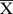, the average value,  = 53%), thus comprising the majority of the pollen assemblages, while that of terrestrial herbs (mainly Poaceae, Cyperaceae, Chenopodiaceae, and *Artemisia*) range from 0% to 39%. Grass pollen varies from 0% to 35%. In addition, concentrations of aquatic herb pollen and fern spores are the lowest as well.

Zone 4 (during the Song Dynasty, 75–85 cm in depth).

The diversity and concentration of terrestrial pollen is at its highest in this zone. The pollen is dominated by trees (up to 72%) including *Castanopsis*, *Pinus* and *Quercus*. *Pinus* pollen appears for the first time in this profile. Besides, Poaceae, Chenopodiaceae, Cyperaceae, *Polygonum*, and *Artemisia* constitute 28% of the pollen assemblage. It is to be noted that *Oryza*-type and grass-type Poaceae pollen (Fig. 3 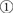,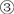), both of which have blurred ornamentation, were found together for the first time. The diameters of *Oryza*-type Poaceae pollen retrieved in our samples range from 40.0 µm to 43.6 µm, while those of the aperture with thick annulus vary from 4.1 µm to 4.6 µm (n = 50). In comparison, grass-type Poaceae is no bigger than 36 µm with a small aperture (about 3.2 µm in diameter) (n = 50). The measurements are in good agreement with that from modern rice (*Oryza sativa*) and wild Poaceae grass [1], [25], [46], [47]. The *Oryza*-type pollen is nearly absent except for this sample in which it reaches 2%. Furthermore, concentrations of aquatic herb pollen and fern spores, e.g. *Typha*, *Myriophyllum*, *Ceratopteris*, *Pteris*, Polypodiaceae and trilete spores, are rather high.

The pollen record, thus describes the vegetation of the mid-late Holocene at the Jiangli site as falling into 4 main episodes: (1) before 7000 cal. BP (before the Majiabang Culture), where the vegetation was grassland dominated by Poaceae, Cyperaceae, and *Artemisia*; (2) from 6000 to 7000 cal. BP (during the Majiabang Culture), where the vegetation was evergreen and deciduous mixed broad-leaved forest dominated by *Castanopsis* and *Quercus*, together with grassland dominated by Poaceae; (3) from 5300 to 6000 cal. BP (during the Songze Culture), where the vegetation was evergreen and deciduous mixed broad-leaved forest dominated by *Castanopsis* and *Quercus*; (4) during the Song Dynasty, where the vegetation was evergreen-deciduous broad-leaved and coniferous mixed forest dominated by *Castanopsis*, *Pinus* and *Quercus*, together with grassland dominated by Poaceae, Chenopodiaceae, and *Artemisia*.

However, due to low concentrations, the phytolith record just consists of some common morphotypes, including elongate psilate, elongate dendritic, and elongate echinate long cells, cuneiform bulliform cells, bilobate, square and rectangular short cells, acicular hair cells, double-peaked *Oryza*-type, bamboo saddles, as well as sponge spicules (Fig. 4). The cuneiform bulliform cells can be identified as *Oryza*-type, reed-type and bamboo-type bulliforms respectively. *Oryza*-type bulliform phytoliths are missing from the parent soil (subsoil) and occur at less than 5% throughout the rest of the profile. On the other hand, bamboo saddles nearly reach 20% during the Songze Culture. Sponge spicules are present in the lowest deposits, indicating a certain amount of marine influence. The climate index reflected by the phytoliths indicates a similar change to that of the vegetation indicated by pollen. In the parent soil, it reaches 2.0 (high sea level), while that in deposits of the Majiabang Culture declines to 0.7–0.8. The index value fluctuates drastically from 0.6 to 1.0 during the Songze Culture, and then rises above 1.0 in the Song Dynasty.

#### Paleoecological data of P02

**Figure 6:**
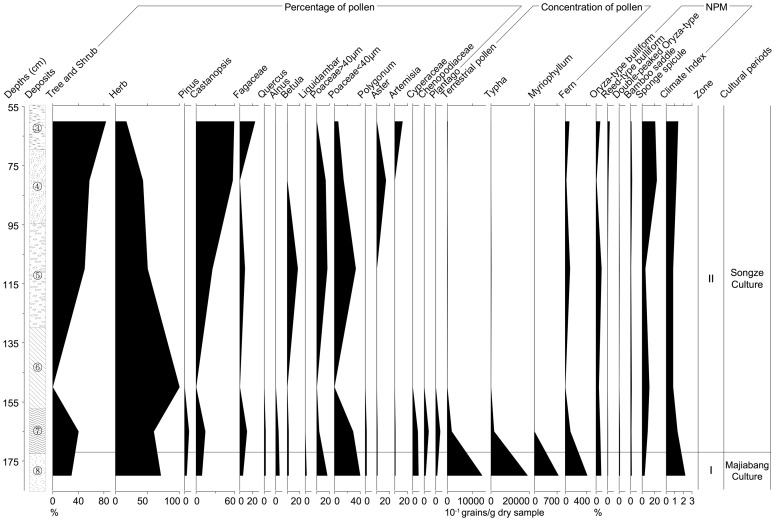
Integrated paleoecological data of P02 from the Jiangli site.

Analysis of the pollen record of TE02N04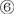 was excluded because of the special constitution of this sample. The scarcity of tree pollen in TE02N04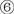 may be attributed to either human impact or climatic conditions. The pollen concentrations and percentages of major taxa provide a basis for dividing the diagram into two pollen zones (Fig. 6):

Zone 1 (about 6000–7000 cal. BP, 172–185 cm in depth).

The concentration of total terrestrial pollen is rather high in this zone. The pollen is dominated by herbs (up to 71%), mainly Poaceae, Cyperaceae. Grass-type and *Oryza*-type Poaceae pollen reach 40% and 17% respectively. While the percentage of arboreal pollen, such as *Castanopsis*, *Alnus*, *Pinus*, *Betula*, *Liquidambar*, *Quercus*, and other Fagaceae reaches 29%. Meantime, concentrations of aquatic herb pollen and fern spores reach high values. For example, *Typha* pollen is represented by more than 1900 grains per gram dry sample.

Zone 2 (5300 to 6000 cal. BP, 55–172 cm in depth).

The concentration of total terrestrial pollen is very low in this zone. The pollen is dominated by trees (ranges from 40% to 83%), e.g. *Castanopsis*, *Betula*, *Alnus*, *Pinus* and *Quercus*. The percentages of *Castanopsis* pollen increase from 14% to 59% (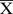 = 39%), representing the majority of the arboreal pollen assemblages. Meantime, that of terrestrial herbs (mainly Poaceae, Cyperaceae and Chenopodiaceae) range from 17% to 60%. Grass-type pollen vary from 6% to 33% (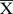 = 21%), while that of *Oryza*-type pollen are from 0 to 17% (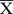 = 9%). In addition, concentrations of aquatic herb pollen and fern spores are rather low as well.

The percentage of *Oryza*-type bulliform phytoliths is also rather low (less than 10%). Sponge spicules, however, are more frequent in the upper deposits. Moreover, the climate index in deposits of the Majiabang Culture is 2.2, while that of the Songze Culture varies from 0.8 to 1.4.

The pollen and phytolith records suggest a similar vegetation and succession from the Majiabang Culture to the Songze Culture to that of profile 01. During this period, the total concentration of terrestrial pollen, aquatic pollen and fern spores decreased drastically while the percentage of arboreal pollen increased gradually. The major vegetation is a combination of evergreen and deciduous mixed broad-leaved forest and grassland. Plants of *Castanopsis* and Poaceae represented the major taxa during the Mid-Late Holocene around the Jiangli site.

#### Paleoecological data of S1–S3

**Figure 7:**
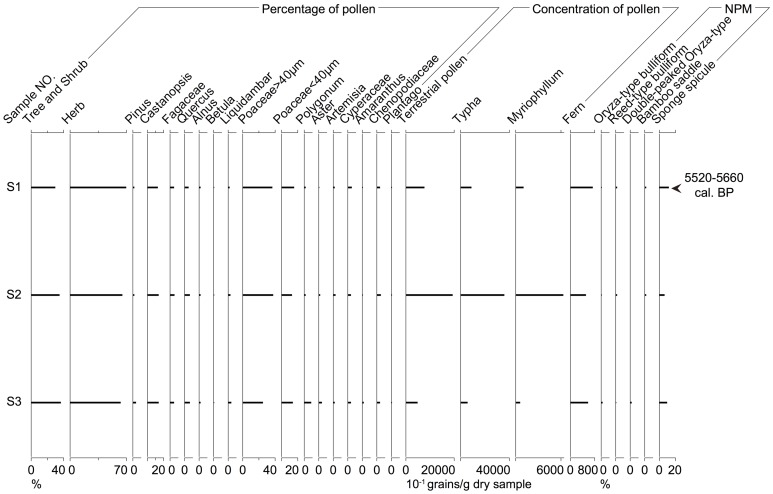
Integrated paleoecological data of rice paddies (S1–S3) from the Jiangli site.

The concentrations of total terrestrial pollen, aquatic herb pollen and fern spores are rather high in each of these three rice paddy deposits (Fig. 7). The pollen of trees and shrubs are about 30% to 37%, while those of herbs range from 63% to 70%, which is the inverse of the situation in P01 and P02. A large amount of *Castanopsis* and *Quercus* pollen (up to 20%), as the major arboreal pollen, suggests the existence of evergreen and deciduous mixed broad-leaved forest in the vicinity of the site. In particular, the average percentage of *Oryza*-type pollen is about 33% compared to that of the grass pollen which is around 14%. Last but not least, the concentration of *Typha* pollen is particularly high, one horizon having almost 4000 grains per gram dry sample.

NPM mainly include phytoliths and sponge spicules. *Oryza*-type bulliform, reed-type bulliform, double-peaked *Oryza*-type and bamboo saddles are identifiable phytoliths. The percentages of *Oryza*-type bulliform phytoliths are particularly low, at only 1%–2%, while those of sponge spicules are around 10%. Besides, the average value of concentrations of *Oryza*-type bulliform is only about 297 (varying from 63 to 765) grains per gram dry sample. All of these NPM suggest a warm and humid wetland landscape, similar to that indicated by the results from pollen analysis.

## Discussion

### Vegetation History at the Jiangli Site during the Mid-late Holocene

Previous studies [48]–[50] have shown that evergreen broad-leaved forest occurred throughout the Taihu Lake region during 7500–5000 BP, which is the equivalent of the environment in the south of Zhejiang today. The annual average temperature was 2–3°C higher than the modern one, while the annual precipitation was 500–600 mm higher than today [51]. Archaeological and environmental research focusing on Holocene lakes and Neolithic sites in the lower reaches of the Yangtze River reveals the development and changes of archaeological culture, climate, and landscape [52]–[59]. Before the Majiabang Culture (7000 cal. BP), the concentration of arboreal pollen retrieved from profile Qidong in the Yangtze River Delta is rather low and the vegetation was dominated by Poaceae and Cyperaceae, which represents salt marsh vegetation [30], [60]. In particular, the environmental landscape of many archaeological sites presented a mixed forest vegetation of evergreen broad-leaved and deciduous broad-leaved forests, and a large expanse of water during the Majiabang Culture, when the development of rice farming was at an initial stage. In the transition to the Songze Culture, fluctuating climate and relatively poor environment led to the development of the Songze Culture itself, while rice farming was subject to certain constraints. During the Liangzhu Culture, however, the climate gradually became cooler and drier, the area of water decreased, and the land area expanded, all of which created excellent environmental conditions for the full development of rice farming.

Xiao’s study suggested that once the sum of *Castanopsis*/*Castanea* and *Cyclobaiamepsis* from cultural deposits in the lower reaches of the Yangtze River reached 10% or over, it may indicate the presence of broad-leaved forest [61]. In the present paper, all but one sample, in which the value is 9%, range from 13% to 75% (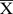 = 37%, STDEVP = 6.5). Pollen analysis shows that pollen of trees and shrubs is the main component of pollen assemblages, among which *Castanopsis*, *Quercus*, *Betula*, and *Liquidambar* constitute the major genera, which indicates that the vicinity of the Jiangli site was covered by evergreen broad-leaved and deciduous broad-leaved mixed forest. The pollen of herbs makes up a certain proportion of the pollen assemblages, among which aquatic plant pollen, such as *Typha* and *Myriophyllum* is dominant, together with fern spores of *Ceratopteris* and *Pteris*, implying there was a large expanse of water nearby. Specifically, the palynological assemblages of the first phase of the Songze Culture were basically similar to those of the late phase of the Majiabang Culture. Evergreen broad-leaved and broad-leaved deciduous mixed forest were present in this region. In the second phase, *Castanopsis* and aquatic plant pollen decreased dramatically, which might have resulted from an intensification of human activities [62], [63]. During the third stage, however, the amount of *Castanopsis* and aquatic plant pollen increased rapidly.

In general, the percentage of pollen of trees and shrubs increased gradually from the late phase of the Majiabang Culture to the Songze Culture, while that of terrestrial herbs decreased. Meanwhile, concentrations of pollen in the Songze Culture were much lower than those in the late phase of the Majiabang Culture. Analysis of charcoal from macrofossils obtained by flotation also confirmed this, and many wetland weeds, such as *Portulaca oleracea*, *Potamogeton* sp., *Rumex* sp., and *Carex* sp., appeared or increased [in proportion] since the Songze Culture [64]. All of these indicate, within a fluctuating climate, a more severe human impact on the local environment and landscape during the Songze Culture, such as stable rice farming in the region.

### Early Agriculture Characteristics at the Jiangli Site

It was considered that *Oryza*-type pollen is characteristized by grain diameters over 36 µm, blurred ornamentation and a large aperture with thick annulus [65], which is in good agreement with the *Oryza*-type pollen (grain diameter >40 µm) we retrieved. In addition, surface pollen analysis in subtropical double-cropping rice areas suggests that if the percentage of Poaceae pollen reached 36% in rice areas, it probably indicates rice agriculture [66]. At the present site, the percentages of Poaceae pollen from rice paddies range from 39% to 53% (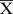 = 48%) which is highly suggestive of rice agriculture activity in paddy fields. Furthermore, compared with profiles (P01 and P02), more *Oryza*-type pollen (grain diameter >40 µm) was retrieved from these rice paddies (S1–S3). The percentages of *Oryza*-type pollen from profile 01 and profile 02 are much lower, while those of grass pollen (grain diameter <40 µm) vary from 0% to 44%. However, these three rice paddies (S1–S3) produced a large quantity of *Oryza*-type pollen (25%–38%), among which the average is about 2.4 times that of the wild grass pollen. Meanwhile, it is worth noting that the percentages of grass pollen are less variable (13%–16%), which implies a more stable arable state and even further development of rice agriculture under human intervention. Last but not least, the average percentage of *Oryza*-type pollen in deposits of the Songze Culture from P02 is about 7 times that from P01, which is considered to be associated with a shorter distance to rice paddies from P02 (about 10 m) than P01 (about 30 m) (Fig. 1). In conjunction with previous studies which suggested that effective dispersal distance of grass pollen, and cultivated crop pollen in particular, is about 60 to 100 m [67]–[69], the tentative conclusion can be drawn that the concentration and percentage of *Oryza*-type pollen are negatively correlated with the distance to the nearest farmland.

However, in comparison with rice pollen, the small percentage and concentration of *Oryza*-type bulliform phytoliths probably suggest the rice paddy was in use for only a very short time, and advances were made in the methods of harvesting the rice (including rice panicles and rice stalks). It is hypothesized by Zhang [70] and ZPICRA [71] that the methods of harvesting crops shifted from obtaining panicles or fruit directly to gathering it as a whole. On the basis of research on rice paddy remains in China and Japan, Udatsu et al. [72] suggested that if the concentration of *Oryza*-type bulliform phytoliths decreased from bottom to top in a continuous sedimentary profile during a cultivated period, it probably could be attributed to harvesting methods changing from cutting panicles to obtaining a whole rice crop. That is to say, if the rice was cut as a whole or the rice paddy was used for a short time, fewer leaves that produce bulliform phytoliths would be incorporated into the soil. In the present paper, pollen identified as belonging to crops (rice) might play an important role in verifying the presence of ancient farmland. Nevertheless, much comparative study is needed to see whether the 40 µm diameter pollen is suitable for distinguishing between crop and non-crop, and whether there are minute distinctions between the pollen of different crops.

Cattail (*Typha*) is an emergent aquatic plant whose pollen is dispersed by wind at relatively high concentrations. Taking this into account, we excluded it when plotting pollen assemblages by percentage. The concentration plot, however, shows that cattail pollen is about 2.5 times more common than herbs in rice paddies, while there is less difference between them in P01 and P02, which demonstrates that cattail was probably grown together with rice during the Songze Culture at the Jiangli site. Cattail was also considered as probably an important crop for food and materials at the Kuahuqiao site [26], [73]. Nevertheless, it is also a kind of weed that caused many problems by invading irrigated agricultural fields (especially rice paddy) [74], drainage channels and ditches [75] in the Old World. Concentration of *Typha* type pollen decreased drastically from the late phase of the Majiabang Culture to the Songze Culture (P01&P02), which might suggest changes in the aquatic environment. Combining these data with the very high concentration of *Typha* pollen from rice paddies (S1–S3), the preliminary conclusion can be made that cattail was probably planted at a high density in the rice paddy and plausibly utilized by the people during the Songze Culture.

Plant macrofossils (such as grain seeds, fruits and epidermis), microfossils (i.e. phytoliths and pollen), as well as historical records and the archaeological context can be used to reconstruct the use of ancient plants and their relationship with humans, especially crops [11]–[13], [15], [24], [41], [42], [64]. However, farmland is the most powerful and best direct evidence for the presence of agriculture. [Being rarely preserved, glebe-farmland (upland field) is hard to identify archaeologically, while rice paddy makes up for this.] Archaeological research on rice paddies can provide information about the structure of the paddy field, farming methods and organization of production, which enrich the methods and techniques of Field Archaeology, while contributing to the reconstruction of ancient paddy field rice farming and its relationship to the development of ancient societies [76].

**Figure 8:**
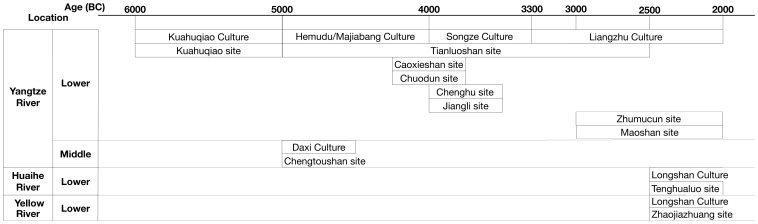
Neolithic rice paddies unearthed or reported in China.

Research on Neolithic rice paddies excavated in China [18], [26], [77]–[84] reveals a possible outline of rice farming, especially the development and spread of rice paddy. The following chart (Fig. 8) illustrates all of the prehistoric rice paddies found or reported in China in chronological order. All of these sites, except for the Kuahuqiao site, exhibited strong physical evidence of prehistoric rice farming in rice paddies, both archaeologically and archaeobotanically. Considering that the expansion of rice agriculture and rapid growth of population, especially during the Late Neolithic in Asia, brought about increased contacts among peoples from different areas [42], [85], cultural interaction between those groups practicing rice agriculture is self-evident. Taking all this evidence into consideration, the preliminary conclusion can be drawn that rice paddy emerged in the middle and lower reaches of the Yangtze River around 7000 BP and probably spread to the lower Huaihe River and the lower Yellow River about 2500 yrs later.

## Conclusions

The biodiversity of plant microfossil assemblages retrieved from profiles 01 and 02, rice paddies (S1–S3) generally suggests a landscape of mixed evergreen-deciduous broad-leaved forest, grassland, and a large expanse of water at or around the Jiangli site during the Majiabang Culture and Songze Culture. The high percentage of Poaceae pollen, especially among which *Oryza*-type pollen is about 2.4 times that of the wild grass one, suggests the presence of rice paddy agriculture at the Jiangli site.

Nevertheless, the duration of rice paddy, field management practices (such as weeding), the specific route taken in the dissemination of rice paddy, and even the discovery of upland fields, etc. are still open questions. We suppose that further studies on changes of Poaceae pollen values in rice paddies and the dissemination of Poaceae pollen in deposits could shed light on these issues.
